# Long-Term Endurance Exercise Training Alters Repolarization in a New Rabbit Athlete’s Heart Model

**DOI:** 10.3389/fphys.2021.741317

**Published:** 2022-02-14

**Authors:** Péter Kui, Alexandra Polyák, Nikolett Morvay, László Tiszlavicz, Norbert Nagy, Balázs Ördög, Hedvig Takács, István Leprán, András Farkas, Julius Gy. Papp, Norbert Jost, András Varró, István Baczkó, Attila S. Farkas

**Affiliations:** ^1^Department of Pharmacology and Pharmacotherapy, Albert Szent-Györgyi Medical School, University of Szeged, Szeged, Hungary; ^2^Department of Internal Medicine, Albert Szent-Györgyi Medical School, University of Szeged, Szeged, Hungary; ^3^ELKH-SZTE Working Group of Cardiovascular Pharmacology, Szeged, Hungary; ^4^Department of Pathology, Albert Szent-Györgyi Medical School, University of Szeged, Szeged, Hungary; ^5^Department of Pharmacology and Pharmacotherapy, Interdisciplinary Excellence Centre, Albert Szent-Györgyi Medical School, University of Szeged, Szeged, Hungary

**Keywords:** athlete’s heart, physical endurance, arrhythmia, ventricular remodeling, biological markers, repolarization abnormality

## Abstract

In the present study, the effect of long-term exercise training was investigated on myocardial morphological and functional remodeling and on proarrhythmic sensitivity in a rabbit athlete’s heart model. New-Zealand white rabbits were trained during a 12-week long treadmill running protocol and compared with their sedentary controls. At the end of the training protocol, echocardiography, *in vivo* and *in vitro* ECG recordings, proarrhythmic sensitivity with dofetilide (nM) were performed in isolated hearts, and action potential duration (APD) measurements at different potassium concentrations (4.5 and 2 mM) were made in the isolated papillary muscles. Expression levels of the slow component of delayed rectifier potassium current and fibrosis synthesis and degradation biomarkers were quantified. Echocardiography showed a significantly dilated left ventricle in the running rabbits. ECG PQ and RR intervals were significantly longer in the exercised group (79 ± 2 vs. 69 ± 2 ms and 325 ± 11 vs. 265 ± 6 ms, *p* < 0.05, respectively). The *in vivo* heart rate variability (HRV) (SD of root mean square: 5.2 ± 1.4 ms vs. 1.4 ± 0.2 ms, *p* < 0.05) and Tpeak-Tend variability were higher in the running rabbits. Bradycardia disappeared in the exercised group *in vitro*. Dofetilide tended to increase the QTc interval in a greater extent, and significantly increased the number of arrhythmic beats in the trained animals *in vitro*. APD was longer in the exercised group at a low potassium level. Real-time quantitative PCR (RT-qPCR) showed significantly greater messenger RNA expression of fibrotic biomarkers in the exercised group. Increased repolarization variability and higher arrhythmia incidences, lengthened APD at a low potassium level, increased fibrotic biomarker gene expressions may indicate higher sensitivity of the rabbit “athlete’s heart” to life-threatening arrhythmias.

## Introduction

Strong physical exercise induces hemodynamic changes in competitive athletes, which leads to adaptive morphological and functional remodeling of the heart described as “athlete’s heart” (O’Keefe et al., 2012). Sports activities improve quality of life and life expectancy, however, a number of tragic sudden cardiac death (SCD) events involving young competitive athletes have been recently reported conveying a devastating emotional impact on families and on the community. While SCD among athletes is rare, approximately 1–2:100,000, it is still 2–4 times more frequent in athletes than in their age-matched controls (Maron et al., 2009).

Numerous pathological anomalies have been associated with SCD in athletes (hypertrophic cardiomyopathy, arrhythmogenic right ventricular cardiomyopathy, etc.), although autopsy findings were inconclusive and normal hearts were demonstrated in 3–6% of the SCD cases among top athletes (Maron et al., 2009). In a review article, Varró and Baczkó proposed a mechanism underlying SCD in athletes that are based on repolarization abnormalities due to potassium channel downregulation, and the concurrent presence of several additional factors, such as cardiac muscle remodeling with the increase of collagen deposits, left ventricular hypertrophy, autonomous nervous system imbalance, genetic defects, electrolyte imbalance (e.g., hypokalemia and hypomagnesemia), certain drugs, doping agents, and dietary ingredients. These factors together can increase the repolarization heterogeneity leading to life-threatening arrhythmias (Varro and Baczko, 2010).

It would be crucial to accurately distinguish benign physiological, morphological, and electrical alterations from those considered to represent potentially serious diseases. The career of athletes is secondary to their lives, although the prevention of the unnecessary termination of career of an athlete and to minimize the risk of SCD are important. However, insights into the electrophysiological features of human athlete’s heart are limited. Thus, it is necessary to obtain further information from appropriate animal models to fortify current knowledge.

As preliminary results, we have recently introduced a long-term endurance training induced rabbit athlete’s heart model, which shares some properties with the human athlete’s heart (Polyak et al., 2018). In the present study, we investigated whether sustained intensive exercise training-induced potentially adverse myocardial morphological and functional remodeling and increased the arrhythmia sensitivity in isolated rabbit “athlete’s hearts.”

## Materials and Methods

### Ethical Statement

Animal maintenance and research were conducted in accordance with the National Institutes of Health Guide for the Care and Use of Laboratory Animals. All procedures using animals were approved by the local ethics committee (including the Ethical Committee for the Protection of Animals in Research at the University of Szeged, Hungary) and conformed to the rules and principles of the 86/609/EEC Directive.

### Experimental Protocol

New Zealand white rabbits from either sex, weighing 3,500–4,000 g, were randomized into sedentary (“Sed,” *n* = 7, 4 male rabbits and 3 female rabbits) and exercised (“Ex,” *n* = 7, 5 male rabbits and 2 female rabbits) groups. “Ex rabbits” underwent a 12-week-long training session, while the “Sed” group did not participate in the training protocol. Running sessions were performed on a self-developed treadmill system, with two separated corridors for the animals and a control panel to modulate speed intensity. The protocol started with a 2-week-long warm-up period, thereafter animals were trained for 5 days/week with 40 min daily running sessions for 12 weeks. The speed intensity of the treadmill was increased progressively and set to 2.5 km/h. Rabbits were supervised continuously throughout the training protocol.

### *In vivo* Echocardiography and ECG

Echocardiography was performed at 0 and 12 weeks of the training protocol. Rabbits were anesthetized with intramuscular ketamine injected into thigh muscle (50 mg/kg). Then, they were shaved at the chest and kept in a supine position on a heating pad. M-mode parasternal long-axis view was applied using an 11.5 MHz transducer (GE 10S-RS, GE Healthcare, Chicago, IL, United States), connected to an echocardiographic imaging unit (Vivid S5, GE Healthcare, Chicago, IL, United States). All parameters were analyzed by an investigator in a randomized and blinded manner. The diameter of ascending aorta, left ventricle (LVID), thickness of the left ventricular posterior wall (LVPW), and interventricular septum (IVS) at systole and diastole were measured in M-mode images. A more extended echocardiography analysis description is shown in Supplementary Material.

After completing the 12-week training protocol, *in vivo* ECG recording was made with needle electrodes that were placed subcutaneously in all four limbs. ECGs were recorded simultaneously with the National Instruments data acquisition hardware (PC card, National Instruments, Austin, TX, United States) and SPEL Advanced Hemosys software (version 3.26, Experimetria Ltd. and Logirex Software Laboratory, Budapest, Hungary).

### Proarrhythmia Protocol in Isolated Hearts

Langendorff-perfusion of the hearts was performed after completion of the *in vivo* ECG at 12th week, as described earlier (Farkas et al., 2006). Briefly, rabbits were anticoagulated with sodium heparin and anesthetized with sodium pentobarbital (∼80 mg/kg) injected into the marginal ear vein. Hearts were rapidly removed *via* thoracotomy and rinsed in ice-cold modified Krebs-Henseleit buffer, which contains (in mM): NaCl 118.5, glucose 11.1, MgSO_4_ 0.5, NaH_2_PO_4_ 1.2, KCl 3, NaHCO_3_ 25, and CaCl_2_ 1.8. The aorta was cannulated and hanged on a Langendorff apparatus. All hearts were retrogradely perfused with modified Krebs-Henseleit buffer for 15 min (“initial perfusion” period), followed by a 30-min perfusion with dofetilide (a selective blocker of the rapid delayed rectifier potassium current) at a concentration of 50 nM (“Dof” period). Our previous examinations showed that dofetilide at this concentration did not provoke Torsades de Pointes (TdP) in healthy isolated rabbit hearts (in contrast to 100 nM) (Farkas et al., 2006), which offered scope for the examination of additional effects that can further increase the incidence of this arrhythmia. In the third period of the protocol, hearts were perfused with modified Krebs-Henseleit buffer again for 30 min to remove the effect of the dofetilide (“washout” period). Volume-conducted ECG was recorded with the National Instruments data acquisition hardware and SPEL Advanced Hemosys software (MDE Gmbh, Heidelberg, Germany).

### Recording Action Potentials in Multicellular Papillary Muscles

Action potentials were recorded at 37°C from the surface of right ventricular papillary muscles using conventional microelectrode techniques. The preparations were mounted in a custom made plexiglass chamber, allowing continuous superfusion with O_2_ saturated Locke solution (containing in mM: NaCl 118.5, KCl 4.5, CaCl_2_ 2.0, MgSO_4_ 1.0, NaH_2_PO_4_ 1.2, NaHCO3 25.0, and glucose 10.0, the pH of this solution was set to 7.35 ± 0.05 when saturated with a mixture of 95% O_2_ and 5% CO_2_) and stimulated with constant current pulses of 1 ms duration at a rate of 1 Hz through a pair of bipolar platinum electrodes using an electrostimulator (Hugo-Sachs Elektronic, modell 215/II, March, Germany). Sharp microelectrodes with tip resistance of 10–20 MΩ, when filled with 3 M KCl, were connected to an amplifier (BioLogic amplifier, model VF 102, Claix, France). The voltage output from the amplifier was sampled using an AD converter (NI 6025, Unisip Ltd, Budapest, Hungary). APD, determined at 25 and 90% level of repolarization, was obtained using Evokewave v1.49 (Unisip Ltd., Budapest, Hungary). After a 30 min of equilibration, 10 consecutive pulses with a cycle length of 400 ms were applied before the hypokalemic (2 mM) Locke solution and the pacing protocol was repeated. Triangulation was calculated as a difference between APD_90_ and APD_25_. Efforts were also made to maintain the same impalement throughout the whole experiment. When the impalement was dislodged, an adjustment was attempted. The measurements were only continued if the action potential characteristics of the re-established impalement deviated less than 5% from the previous one.

### Measurement of the ECG Intervals *in vivo* and *in vitro*

After the 12 weeks training protocol under ketamine (50 mg/kg im.) anesthesia, 20 min of ECG was registered with four limb needle ECG electrodes. *In vivo* ECG intervals were measured at the 10th min after initiation of the recording according to Farkas et al. (2004). *In vitro* ECG parameters of the Langendorff perfused rabbit hearts were evaluated in sinus rhythm 1 min before the end of “initial perfusion” period and 10 min after the start of the “Dof” period (as shown in Supplementary Material). RR, PQ, QRS, QT, and Tpeak-Tend (interval between the peak and end of the T wave) intervals were measured by manual positioning on screen markers of 40 consecutive sinus beats, and mean values were calculated. The QT interval was defined as the time from the first deviation from the isoelectric line during the PR interval until the end of the T wave. Where the T wave overlapped the following P wave or the QRS complex of the subsequent beat, the extrapolation method was applied to measure the length of the QT interval, that is, the end of the T wave was extrapolated from the curve of the T wave to the isoelectric line under the P wave or the QRS complex. Since the QT interval is influenced by the heart rate, the rate corrected QT interval (QTc) was calculated with a correction method described earlier (Farkas et al., 2009; Polyak et al., 2018). Baseline data in sinus rhythm for QT intervals together with the corresponding RR intervals were obtained from *in vitro* and *in vivo* pooled ECG data of our laboratory (containing hundreds of *in vitro* and *in vivo* rabbit experiments). Rate corrected QT interval (QTc) was calculated both at *in vivo* [QTc_n_ = QT_n_–0.354 ⋅ (RR_n–1_ − 295)] (Polyak et al., 2018) and *in vitro* [QTc_n_ = QT_n_–0.326 ⋅ (RR_n–1_ − 404)] (Farkas et al., 2009) using formulas developed by our laboratory.

The T_peak_-T_end_ interval was measured according to Antzelevitch and Oliva (2006) in the standard limb Lead II of the ECG. When the end of the T_peak_-T_end_ interval overlapped the following P wave, the extrapolation method (Farkas et al., 2004) was applied.

#### Measurement of the Beat-to-Beat Variability and Instability of the ECG Intervals in Sinus Rhythm

Beat-to-beat variability and instability (BVI) parameters of the RR, PQ, QRS, QT, and Tpeak-Tend intervals were calculated from 40 consecutive sinus beats at the same time points where mean ECG intervals were measured according to Farkas et al. (2009) and Orosz et al. (2014). A computer program was developed in a.NET environment to obtain the BVI parameters. The calculation of BVI parameters is summarized in Supplementary Material.

### Arrhythmia Analysis

*In vitro* ECG recordings were replayed offline and arrhythmia incidences were calculated throughout the whole experiment (“initial perfusion,” “dofetilide period,” and “wash-out period”). Ventricular tachyarrhythmia definitions of Lambeth Conventions I (Walker et al., 1988) were applied together with all other (non-tachyarrhythmia) ventricular arrhythmia definitions of Lambeth Conventions II (Curtis et al., 2013). Runs of 4 or more consecutive ventricular premature beats without the TdP-like twisting QRS morphology were differentiated from TdP and were defined as ventricular tachycardia (Farkas et al., 2009). The total number of arrhythmic beats was calculated as the sum of all ventricular premature beats (i.e., arrhythmic beats in any kind of arrhythmia).

### Histology and Morphometry

Total cardiac mass was measured after finishing the Langendorff protocol, then the atria were removed from the hearts, and ventricles were weighed to calculate heart-weight-to-body weight and ventricular-weight-to-body weight ratio.

Samples were taken from the left ventricular free wall for histological studies. Heart sections were stained with Crossman-trichrome to identify collagen deposition. Semiquantitative analysis was performed by a pathologist to score the degree of the interstitial fibrosis with the following criteria: 0 = negative; 1 = mild; and 2 = moderate. The pathologist was blinded to the training protocol of the animals.

### Real-Time Quantitative PCR of Gene Expressions of Fibrotic Biomarkers and I_Kr_ and I_Ks_

RNA was isolated from left ventricular free wall samples with the Direct-zol RNA MiniPrep (Zymo Research, Irvine, CA, United States, Cat. No. R2051). cDNA molecules were synthesized from mRNA templates by reverse transcription, using High-Capacity cDNA Reverse Transcription Kit (Applied Biosystems, Waltham, MA, United States, Cat. No. 4368814). Real-time PCR was conducted with gene-specific primers and SYBR green (Thermo Fisher Scientific, Waltham, MA, United States, Cat. No. K0222) on ABI PRISM^®^ 7000 Sequence Detection System (Thermo Fisher Scientific, Waltham, MA, United States, Cat. No. K0222). After each run, a melting point analysis was performed by measuring fluorescence intensity. The expression levels of fibrotic biomarker encoding genes were examined (transforming growth factor-β1, TGF-β; fibronectin-1, FN-1; pro-alpha1 chain of type I collagen, COL1A1; pro-alpha1 chain of type III collagen, COL3A1; matrix metalloproteinase-2, MMP-2; and tissue inhibitor of metalloproteinase-1, TIMP-1), as well as ion channel subunit expression underlying the rapidly and the slowly activating component of I_K_ channel: I_Kr_ (KCNH2) and I_Ks_ (KCNQ1), respectively. We calculated the relative copy numbers of mRNAs by normalizing each cDNA to the geometric average of β-actin (ACTB), signal-recognition-particle assembly 14 (SRP14), and ribosomal protein S5 (RPS5) expression.

### Statistical Analysis

Continuous data were expressed as mean ± SEM. Independent variables from echocardiographic parameters, ECG intervals, BVI parameters, arrhythmia numbers, and fibrosis scores were compared with Mann–Whitney *U*-test as independent variables between the groups. The repeated measure of ANOVA was applied to compare the prolongation of the QTc interval. Relative gene expression was evaluated by using *t*-test type 3. *p* < 0.05 was taken as indicative of a statistically significant difference between values.

## Results

### Echocardiography

At the end of the protocol, endurance training resulted in significantly greater internal end-diastolic diameter of the left ventricle (LVIDd) (Table 1). The thickness of the LVPW and the IVS did not differ between the groups neither in systole nor in diastole (Table 1). Thus, the left ventricle became dilated due to the endurance training without ventricular hypertrophy. The diameter of the aorta became significantly greater in the “Ex” group compared with that in the “Sed” group. After long-term training, ejection fraction and fractional shortening did not differ between the “Ex” and “Sed” groups. The complete list of measured echocardiographic parameters is shown in Table 1.

**TABLE 1:** Echocardiography parameters.

	Before training protocol			After training protocol		
	“Sedentary” group	“Exercised” group	*p*-value	“Sedentary” group	“Exercised” group	*p*-value
IVSd, mm	3.28 ± 0.16	3.18 ± 0.15	0.646	3.24 ± 0.21	3.03 ± 0.16	0.437
IVSs, mm	4.69 ± 0.15	4.79 ± 0.05	0.525	4.75 ± 0.23	4.20 ± 0.24	0.129
LVIDd, mm	14.70 ± 0.54	15.80 ± 0.32	0.104	14.44 ± 0.62	17.25 ± 0.31	0.002*
LVIDs, mm	10.58 ± 0.36	10.84 ± 0.21	0.537	10.57 ± 0.58	11.81 ± 0.29	0.078
LVPWd, mm	3.13 ± 0.13	3.07 ± 0.13	0.141	3.13 ± 0.20	2.94 ± 0.13	0.447
LVPWs, mm	4.74 ± 0.24	4.95 ± 0.27	0.577	4.59 ± 0.22	4.96 ± 0.42	0.447
Ao, mm	8.64 ± 0.29	9.02 ± 0.28	0.374	7.98 ± 0.16	9.09 ± 0.41	0.027*
EF,%	64.29 ± 3.09	61.57 ± 1.85	0.466	57.43 ± 3.17	64.29 ± 2.47	0.113
FS,%	32.43 ± 2.42	30.43 ± 1.25	0.477	27,86 ± 2.04	32.71 ± 1.7	0.092

*The effect of exercise training on echocardiographic cardiac dimensions and performance values were measured before (at 0th week, control measurements) and after (at 12th week) the training protocol.*

*IVSs and IVSd, interventricular septum in systole and diastole; LVIDs and LVIDd, left ventricular internal diameter in systole and diastole; LVPWs and LVPWd, left ventricular posterior wall in systole and diastole; Ao, aortic root diameter; EF, ejection fraction; FS, fractional shortening.*

All values are means ± SEM. *P < 0.05 vs. ‘Sedentary’ (embolded in the text).

### ECG Intervals *in vivo* and *in vitro*

**FIGURE 1:**
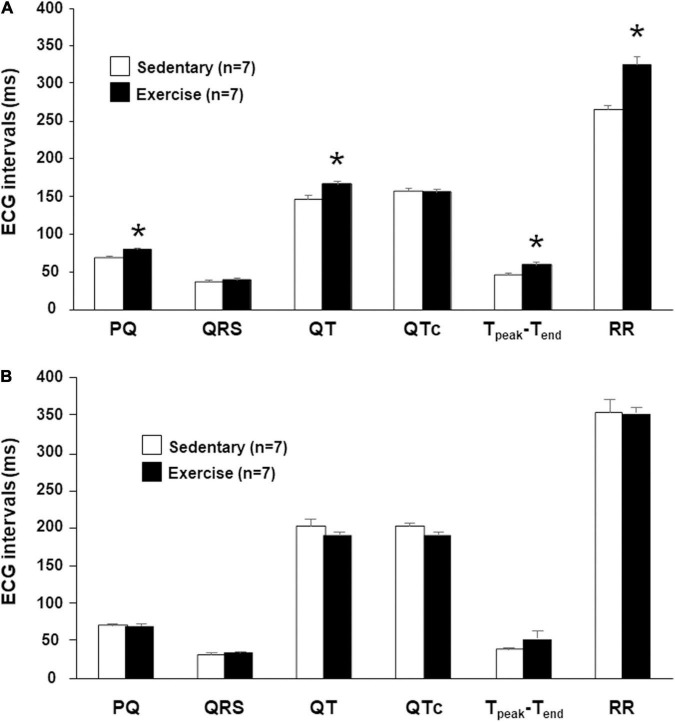
The mean *in vivo* **(A)** and *in vitro* **(B)** ECG intervals at 12th week. Values were derived from 40 consecutive ventricular complexes during sinus rhythm. All values are shown as mean ± SEM. *p < 0.05 vs. “Sedentary.”

The intervals PQ and RR were significantly longer in running rabbits possibly indicating an increased vagal tone. QT and T_peak_-T_end_ intervals were significantly longer in the “Ex” group compared with those measured in the control rabbits. However, the heart rate corrected QT (QTc) values did not differ between the “Ex” and “Sed” groups indicating a frequency dependency of the length of repolarization. There was no significant difference in the QRS intervals, thus the ventricular depolarization was not affected by the intensive training (Figure 1A).

In isolated perfused hearts, the heart rate (RR interval) and the atrioventricular propagation time (PQ interval) were not different in the two groups (Figure 1B). Therefore, increased parasympathetic tone disappeared in the “Ex” group due to the denervation. No significant differences were found in any baseline ECG intervals at the beginning of the *in vitro* proarrhythmia protocol (Figure 1B).

### Beat-to-Beat Variability and Instability Parameters *in vivo*

**FIGURE 2:**
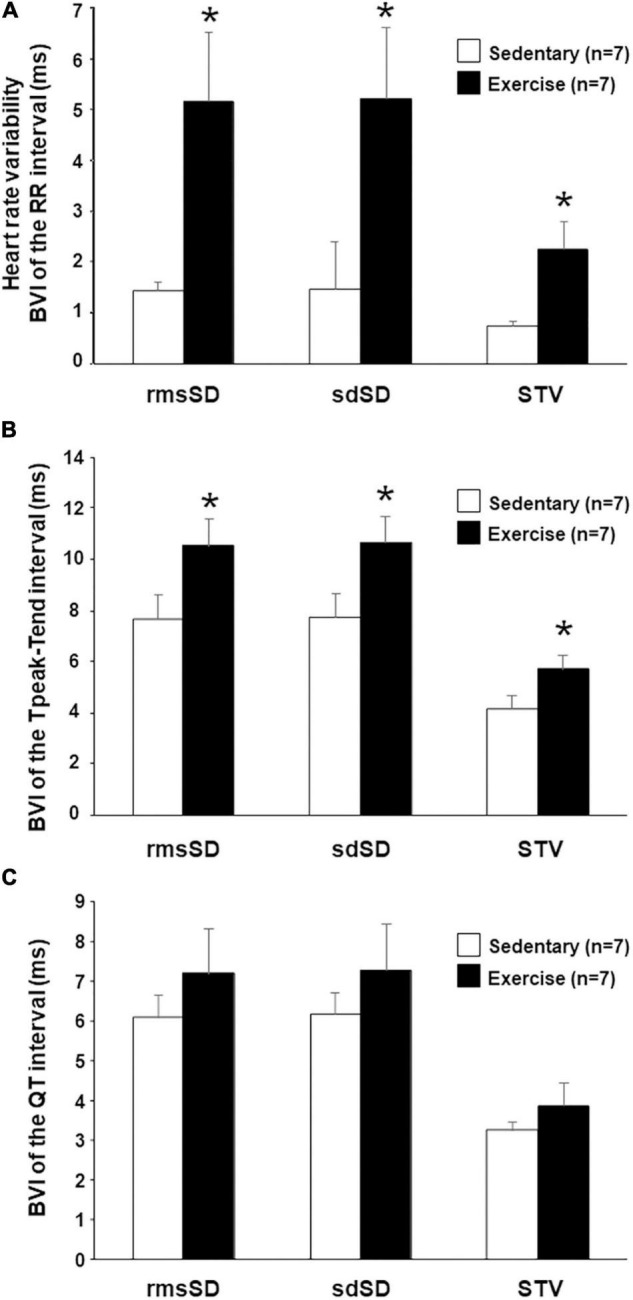
*In vivo* beat-to-beat variability parameters (BVI) of the RR interval **(A)**, T_peak_-T_end_ **(B)**, and QT intervals **(C)** at 12th week. RMS, root mean square; rmsSD, root mean square of successive differences; sdSD, SD of successive differences; STV, short-term variability. All values are shown as mean ± SEM. *p < 0.05 vs. “Sedentary.”

Most of the calculated BVI parameters of the RR intervals (heart rate variability [HRV]) showed a marked and significant increase in running rabbits in comparison with the control group (Figure 2A), which indicates an increased vagal tone in the exercised animals. Analysis of repolarization variability parameters showed a significant increase in all the T_peak_-T_end_ BVI parameters in trained animals compared with the sedentary group (Figure 2B). Interestingly, no significant differences were found between the “Ex” and “Sed” groups regarding QT variability (Figure 2C).

### Beat-to-Beat Variability and Instability Parameters *in vitro*, the Effect of Dofetilide

Importantly, the HRV parameters (RR variability parameters) markedly decreased when the hearts were removed and Langendorff perfused, which indicates the cessation of parasympathetic tone (Table 2).

**TABLE 2:** Heart rate variability parameters.

	*In vivo*			*In vitro*		
	“Sedentary” group	“Exercised” group	*p*-value	“Sedentary” group	“Exercised” group	*p*-value
Mean_RR_, ms	265.5 ± 5.7	324.5 ± 10.5	0.001*	354.2 ± 17.9	352.5 ± 8.3	0.930
SD_RR_, ms	1.5 ± 0.2	4.4 ± 0.8	0.026*	1.6 ± 0.7	3.4 ± 1.7	0.360
RMS_RR_, ms	265.5 ± 5.7	324.5 ± 10.5	0.001*	354.2 ± 17.9	352.5 ± 8.3	0.931
rmsSD_RR_, ms	1.4 ± 0.2	5.2 ± 1.4	0.035*	2.2 ± 1.2	5.0 ± 2.8	0.401
sdSD_RR_, ms	1.5 ± 0.2	5.2 ± 1.4	0.047*	2.2 ± 1.2	5.1 ± 2.8	0.401
STV_RR_, ms	0.7 ± 0.1	2.2 ± 0.5	0.016*	0.7 ± 0.2	2.0 ± 0.9	0.199
LTV_RR_, ms	1.4 ± 0.2	3.4 ± 0.8	0.049*	1.0 ± 0.3	2.0 ± 0.8	0.317
TI_RR_, ms	1.8 ± 0.2	4.1 ± 1.0	0.047*	1.0 ± 0.2	2.3 ± 0.5	0.032*

*Heart rate variability (HRV) parameters in vivo and in vitro.*

*RMS, root mean square; rmsSD, root mean square of successive differences; sdSD, SD of successive differences; STV, short-term variability; LTV, long-term variability; TI, total instability.*

All values are means ± SEM. *P < 0.05 vs. ‘Sedentary’ (embolded in the text).

**FIGURE 3:**
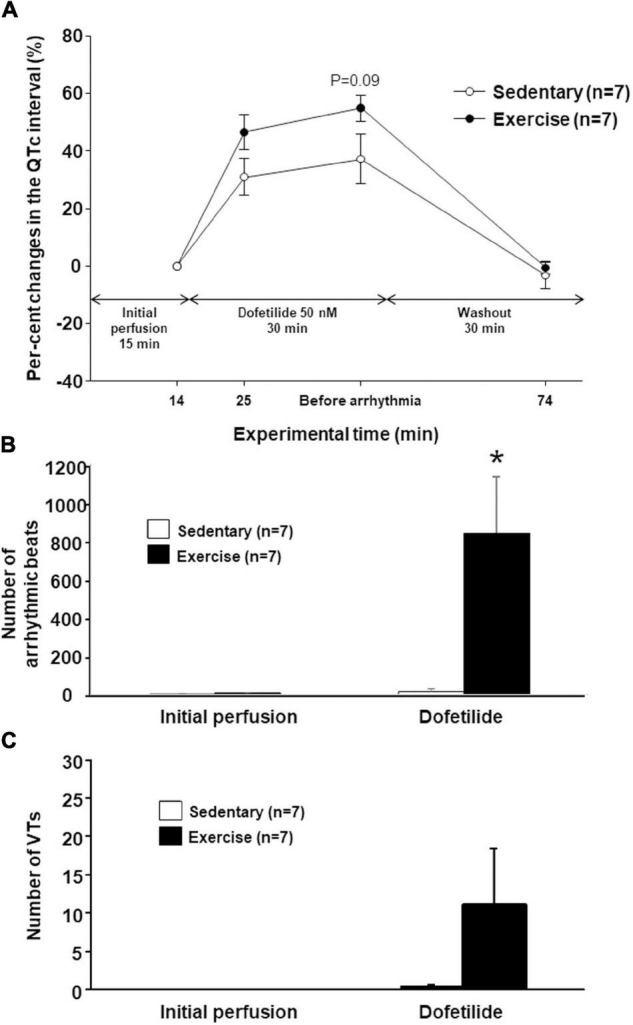
The percentage change in the mean heart rate-corrected QT (QTc) interval under dofetilide challenge *in vitro* in the last minute before dofetilide administration (14th min), 10 min after the start of dofetilide infusion (25th min), immediately before arrhythmia occurred, and after “Washout” (74th min) **(A)**. The total number of arrhythmic beats **(B)** and the number of ventricular tachycardia episodes **(C)** under dofetilide challenge *in vitro*. All values are shown as mean ± SEM. *p < 0.05 vs. “Sedentary.”

The I_Kr_ inhibitor dofetilide at a concentration of 50 nM was applied to test the sensitivity of repolarization of the rabbit athletes’ hearts in the Langendorff preparation, *in vitro*. Dofetilide markedly increased the QTc interval in the hearts of “Ex” and “Sed” groups too, however, the QTc prolongation was more pronounced in the exercised hearts (Figure 3A). Mean values of the PQ, QRS, and T_peak_-T_end_ intervals did not differ significantly between the groups in either experimental period (data not shown).

No significant difference was found in the BVI parameters of any ECG intervals between the “Ex” and “Sed” groups at the beginning and at the end of the initial drug-free perfusion. Dofetilide perfusion tended to increase the QT and T_peak_T_end_ variability parameters in the hearts of “Ex” and “Sed” groups too, but no significant differences were found in these parameters between the groups (data not shown).

### Arrhythmia Susceptibility Analysis in Isolated Hearts

The number of arrhythmic beats did not differ between the “Ex” and “Sed” groups during drug-free “Initial perfusion.” However, dofetilide perfusion significantly increases the number of arrhythmic beats in the “Ex” group as compared with the “Sed” group (Figure 3B). The previous endurance training had a tendency to increase the number or ventricular tachycardias during dofetilide perfusion in the hearts of the “Ex group,” but this parameter did not differ significantly between the “Sed” and “Ex” group (Figure 3C). TdP ventricular tachycardia did not develop during dofetilide perfusion in any groups. There was no significant difference in any arrhythmia parameters between the groups during the washout period (data not shown).

### Action Potential Duration Measurements in Multicellular Papillary Muscle Preparations

**FIGURE 4:**
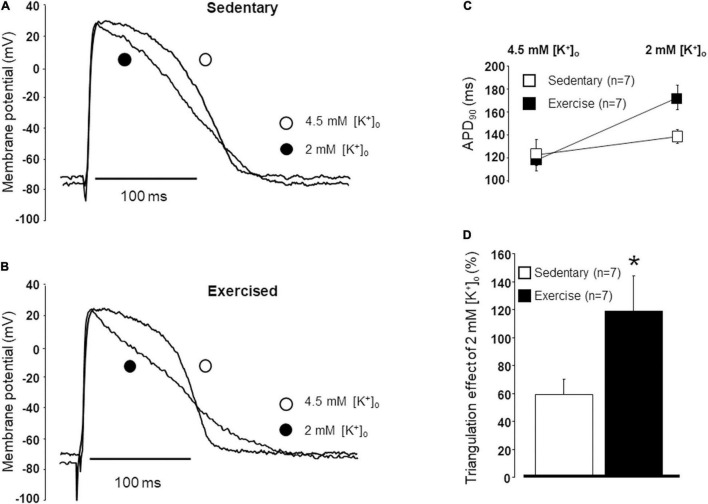
Representative examples of the action potentials in papillary muscles obtained from hearts excised from sedentary **(A)** and exercised **(B)** animals under normal and low potassium levels. The duration of the action potential measured at 90% repolarization (APD_90_) in papillary muscles obtained from hearts excised from sedentary and exercised animals under normal (4.5 mM) and low (2.0 mM) potassium levels **(C)**. Triangulation measured in papillary muscles obtained from hearts excised from sedentary and exercised animals under low potassium levels **(D)**. All values are shown as mean ± SEM. *p < 0.05 vs. “Sedentary.”

The durations of the action potentials were compared in papillary muscles obtained from exercised and sedentary animals under normal and lower potassium levels (Figures 4A,B). No difference was found in the APDs between the “Ex” and “Sed” groups under normal potassium (4.5 mM) level (Figure 4C). However, under low potassium (2.0 mM) circumstances, the APD was significantly longer in exercised papillary muscles than that measured in the sedentary ones (Figure 4C). Also, when low potassium solution was applied, the rate of triangulation was significantly larger in the exercised group than that measured in the sedentary group (Figure 4D).

### Relative Gene Expression Analysis

**FIGURE 5:**
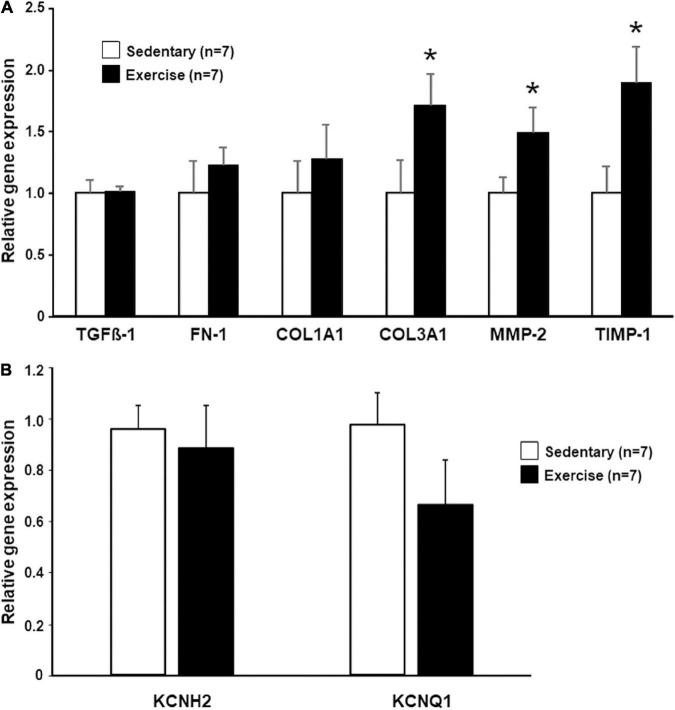
The relative expression levels of different fibrosis synthesis and degradation biomarker genes **(A)** and I_Kr_- and I_Ks_-related genes **(B)** determined by real-time quantitative PCR (qRT-PCR). The mRNA levels were quantified in tissue samples collected from the left ventricle (LV). The data were normalized to the expression level of β-actin (ACTB), SRP14, and RPS5, and are presented as mean ± SEM. *p < 0.05 vs. “Sedentary.”

After 12-week of endurance training, the relative gene expression of fibrosis synthesis and degradation biomarkers of the left ventricle free wall was assessed. The level of mRNA expression of COL3A1, MMP-2, and TIMP-1 were significantly increased in the “Ex” group as compared with that measured in the “Sed” group. COL1A1 and FN-1 expression showed a moderate, but not significant increase in the exercised group (Figure 5A). The expression level of TGF-ß was similar in the “Ex” and “Sed” groups (Figure 5A).

The *KCNH2* (hERG, the main pore-forming subunit responsible for I_Kr_) gene was expressed at approximately the same level in the “Ex” and “Sed” groups (Figure 5B). The expression level of the I_Ks_-related channel encoding *KCNQ1* was slightly lower in the exercised hearts, but the difference proved to be not statistically significant between the “Ex” and “Sed” groups (Figure 5B).

### Morphometry and Histology

**FIGURE 6:**
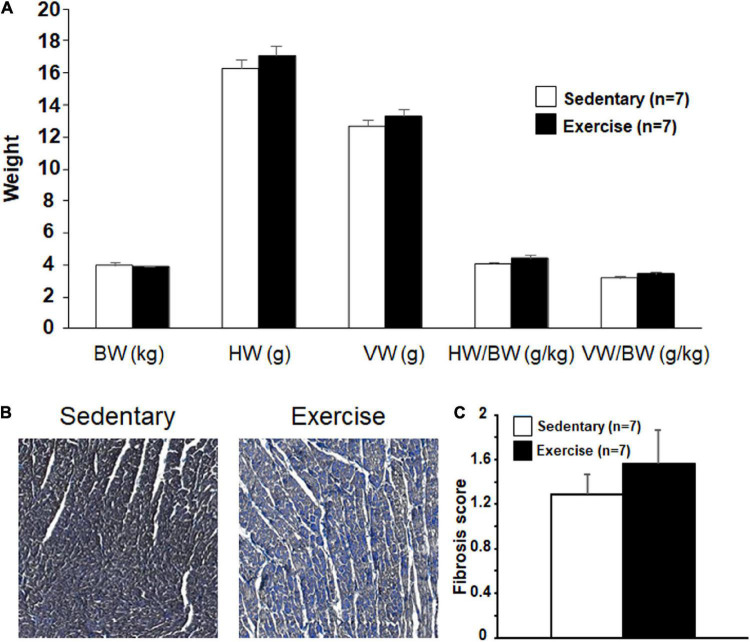
The mean values for body weight (BW), heart weight (HW), ventricular weight (VW), HW/BW, and VW/BW ratio at 12th week **(A)** and representative Crossman’s trichrome stains taken from histological sections of “Sedentary” and “Exercised” left ventricle **(B)** and the mean microscopic fibrosis scores for “Exercised” and “Sedentary” groups **(C)**. Pronounced interstitial fibrosis **(A)** and slightly increased fibrosis score **(B)** are present in the “Exercised” group. Heart weight and ventricular weight are in g and body weight in kg. All values are shown as mean ± SEM.

The total heart weight, ventricular weight, and their ratios to body weight were determined after the completion of the Langendorff perfusion protocol. The long-term exercise increased these parameters (except bodyweight), however, the difference was not significant (Figure 6A).

To assess the level of collagen deposition in the left ventricle, Crossman’s trichrome staining was performed. Similarly, to the relative gene expression results, semiquantitative score analysis showed an increased level of fibrosis in the “Ex” group compared with that in the “Sed” group (Figures 6B,C).

## Discussion

This study is the extension of our earlier long-term endurance training-induced rabbit athlete’s heart model study (Polyak et al., 2018) giving more insights into the electrophysiological properties of the model. Long-term physical exercise decreased the resting heart rate and increased the HRV, which indicates an increased parasympathetic tone as a result of long-term training. Morphological adaptations, such as left ventricle end-diastolic and relative aortic diameter dilation were found, which was accompanied by a close to significant left ventricular fibrosis together with a significantly increased fibrotic gene expression.

Additionally, the present study tested the proarrhythmic sensitivity of isolated rabbit athlete’s hearts. An increased arrhythmic beat activity and a close to significant QTc prolongation were observed in exercised isolated hearts challenged with the I_Kr_ inhibitor dofetilide. In addition, papillary muscles from exercised hearts were more sensitive to the APD-prolonging and triangulation-increasing effects of dofetilide under low potassium concentrations. These results imply an increased proarrhythmic sensitivity in the exercised rabbit hearts. Based on these results, our rabbit animal model with the applied training protocol may be a useful experimental model for further investigations of the cardiovascular effects of long-term physical exercise.

### A Rabbit Model to Mimic the Human Athlete’s Heart

To our knowledge, our recently introduced rabbit model is the first to mimic a human athlete’s heart, especially on the cardiac electrophysiological level (Polyak et al., 2018). Radovits et al. (2013) developed a rat training model where swimming-induced left ventricular hypertrophy was found by echocardiography and histomorphometry. Chen et al. (2000) and Benito et al. (2011) applied a treadmill system for long-term endurance training of rats. It must be emphasized that mouse and rat have distinctly different potassium channel expression patterns than other mammals including humans. Rat and mouse myocytes lack functional sarcolemmal I_Kr_ and I_Ks_ potassium currents (Tamargo et al., 2004), thus repolarization-related arrhythmias cannot be examined in these species, and results cannot be extrapolated to humans. Some acute and chronic endurance training rabbit models have been described, although less of them investigated the cardiovascular effects of exercise, especially on the electrophysiological level (Lozano et al., 2020). Gaustad et al. (2010) tested a VO2max protocol in rabbits and found that rabbit is a suitable species for studying responses to training and could be of great importance for showing novel cellular cardiac adaptations for training. Hexeberg et al. (1995) found a structural myocardial remodeling and increased contractile reserve after a 10-week exercise training program on rabbits. They concluded that rabbits can be used to study the myocardial effects of endurance training. Considering the ion channel kinetic properties, the I_Ks_ measured in dog (Liu and Antzelevitch, 1995) and rabbit ventricles (Lengyel et al., 2001) best resemble that measured in human hearts. In this respect, the present study investigated the effects of endurance exercise training on cardiac remodeling in the rabbit that shares similar cardiac electrophysiological and autonomic neural properties with humans.

Based on recent studies, the treadmill running model seems to be the preferred option to investigate the effect of chronic physical exercise since it allows uniform and well-controlled exercise workloads (Hoydal et al., 2007). Previous studies demonstrated that rabbits could obtain a documented cardiovascular training effect by using the proper intensity, duration, and frequency of exercise (DiCarlo and Bishop, 1990; Liu et al., 2000). Carroll and Kyser (2002) used a rabbit model to determine the effects of exercise training during the development of obesity, by 12-week-long treadmill protocol at 18–20 m/min (1.2 km/h) of maximum speed and 50–60-min daily running sessions. Exercise-trained rabbits had slower resting heart rates in both lean and obese animals (Carroll and Kyser, 2002). Such et al. (2008) found lower resting heart rates and longer ventricular effective refractory periods at similar workload in rabbits.

The applied running protocol in our model made the participating rabbits physically tired and sometimes exhausted. As the New Zealand white rabbit is a relatively physically inactive species, this workload is thought to be convenient to mimic regular, high-intensity human training activity.

### Morphological Adaptations of the Cardiovascular System to Long-Term Exercise

Strongly related to the type of sports activity, Morganroth et al. (1975) arbitrarily classified the athletic endeavors into “isometric” and “isotonic” varieties, based on the presumptive hemodynamic stresses of the two types of activity. In “isotonic exercise” activities (e.g., long-distance running, cycling, and swimming), the change in LV architecture might be considered a form of eccentric remodeling in that both chamber size and LV mass are increased, similar to those in chronic volume overloads (Kim and Baggish, 2016). Recently, D’Andrea et al. (2010b) reported significantly greater LV end-diastolic diameter in endurance-trained athletes than in the strength-trained athletes and control participants.

Similar to these earlier findings in athletes, increased cardiac LV end-diastolic diameter was detected in the exercised rabbit group, which indicates a structural response to the increased cardiac volume load and corresponds to the effect of long-term endurance training. Our endurance-trained rabbits presented a relatively greater aortic root diameter compared with the control group, which implies the effect of cardiac volume overload in these exercised animals. These results are in a good accordance with the results of a large cohort study of competitive athletes, where male endurance athletes showed greater aortic and LV cavity dimensions than sex-matched strength athletes (Pelliccia et al., 1999). Significantly greater aortic root diameter was found in elite strength-trained athletes (D’Andrea et al., 2010a). Neither IVS, nor posterior wall thickening was detected, LV contractile function (EF and FS) remained normal in this model that represents a physiologic adaptation to vigorous exercise training. Similarly, Such et al. (2008) did not observe differences between sedentary and exercised rabbit hearts in terms of hypertrophy. However, endurance-trained athletes may show LV wall thickening, parallel with the extreme LV dilatation (Pelliccia et al., 1999). The reason for different echocardiography results concerning hypertrophy might be the outcome of the different hemodynamic and loading conditions of the heart depending on various training activities. This indicates that myocardial adaptations may depend on the cardiac pressure loads and exercise-related cardiac blood volumes in different sports activities (Hoogsteen et al., 2004).

### Cardiac Fibrotic Changes and Lower I_Ks_-Related Gene Expression Associated With Long-Term Physical Exercise

Myocardial extracellular matrix (ECM) is a complex microenvironment containing a large portfolio of matrix proteins, signaling molecules, proteases, and cell types that play a fundamental role in the myocardial remodeling process (Spinale, 2007). Fibrosis and inflammatory infiltrates have been identified in well-trained athletes (Tahir et al., 2017) and forced swimming rats (Chen et al., 2000). Benito et al. (2011) demonstrated an increase in atrial and ventricular inflammation and fibrosis and a greater risk of ventricular arrhythmias in the “marathon rats” after treadmill exercise. Our significantly increased MMP-2 and TIMP-1 gene expressions, which play an important role in the degradation of cardiac ECM (Brown et al., 2005), indicate higher fibrotic activity in exercised hearts. The balance of MMPs and TIMP determine the maintenance of interstitial tissue homeostasis. Similarly, to our results, increased levels of MMP-2 and TIMP-1 were found in rats after acute exhaustive swimming (Olah et al., 2015). Polyakova et al. (2011) found higher levels of Collagen type I and II, TIMP1, MMP2 in explanted human hearts with heart failure due to either dilated, ischemic or inflammatory cardiomyopathy (myocarditis) as compared with controls. Collagen type I and collagen type III are the two major components of the cardiac ECM (Polyakova et al., 2011). The relative gene expression of type III collagen in our study was significantly greater in the exercised group in our study. Similar results were found in cardiac remodeling in heart failure (Zannad et al., 2010) and in spontaneous hypertensive rats, where the ratio of collagen type I/III was decreased after 10 weeks compared with normotensive control rats, meanwhile the collagen concentration did not differ between the groups (Mukherjee and Sen, 1990). Since collagen type III is a major component of the cardiac ECM, its qualitative-quantitative changes may have an important role in cardiac pathophysiology. The higher collagen synthesis promotes higher expression of TIMP and MMP. The histological analysis of the hearts of the rabbits in our study implies that long-term exercise-induced fibrosis in the myocardium. These histology results were strongly corroborated by our gene expression data. The collagen deposition and consequent myocardial fibrosis is a result of an imbalance of synthetic/degradative events. Fibrotic changes affect impulse conduction in the heart, and thus fibrosis may increase the risk of the development of ventricular arrhythmias (Morita et al., 2014).

It was postulated in some reviews that potassium channel downregulation may be present in an athlete’s heart (Varro and Baczko, 2010). The current study has examined the most common long QT syndrome-susceptibility genes encoding key ion channel subunits *KCNQ1* (LQT1) and *KCNH2* (LQT2). We found slightly decreased I_Ks_-related channel *KCNQ1* levels in the exercised hearts. I_Ks_ downregulation accompanied by an incorrect QT adaptation may result in higher arrhythmic risk especially during sympathetic stress (i.e., high-level exercise) (Tseng and Xu, 2015). I_Ks_ downregulation might contribute to the higher dispersion facilitating the development of reentry circuits (Varro and Baczko, 2010). Interestingly, I_Ks_ downregulation was found in the chronic AV blocked dog model, which shares some similar properties with the athlete’s heart in terms of cardiac adaptation (Volders et al., 1999). In addition, hypokalemia is known to increase I_Ks_ and decrease I_Kr_ (Varro et al., 2021). Therefore, the application of dofetilide which inhibits I_Kr_ can produce a more robust effect on repolarization in case I_Ks_ is downregulated and thereby the hypokalemia cannot properly compensate for the loss of I_Kr_ density.

### Exercise Training Increased Parasympathetic Activity

Reduced heart rate and HRV, i.e., the beat-to-beat variability of the cycle length (RR interval) in sinus rhythm *in vivo*, are considered parameters of parasympathetic activity (Aubert et al., 2003). Accordingly, exercise training elicited significant reductions in baseline heart rate accompanied by significant increases in almost all examined sinus HRV parameters in our running rabbits. With regards to atrial electrophysiological properties, exercise training increased PQ interval (atrioventricular conduction time) *in vivo*. These results correlate well with the increased time-domain indices of HRV presented in competitive athletes (Langdeau et al., 2001). Studies using a canine model of sudden death demonstrate that endurance exercise training (treadmill running) enhanced cardiac parasympathetic regulation (increased heart rate variability) and protected against ventricular fibrillation induced by acute myocardial ischemia (Billman, 2009). Bilateral vagotomy prevents the development of TdP in rabbits *in vivo* (Farkas et al., 2008). Furthermore, bradycardia in the absence of increased vagal tone contributes to the arrhythmogenesis. Thus, the increased vagal tone and bradycardia might have importance in the arrhythmia development.

Recently, Boyett et al. (2013) have shown that training-induced bradycardia in rats resulted from the reduced expression of the *HCN4* gene, i.e., downregulation of the channels conducting the pacemaker I_f_ current rather than as a consequence of changes in the cardiac autonomic regulation. In our study, exercise training might have had only a minor effect on intrinsic heart rate since heart rate and HRV differences disappeared in isolated hearts. The difference between the results can be accounted for methodological and species differences. Since we have not investigated *HCN4* expression in our present study, some downregulation of I_f_ in our model cannot be ruled out.

### Ventricular Repolarization in the Rabbit Athlete’s Heart

Previous studies found longer QTc in endurance athletes compared with age-matched controls (Lengyel et al., 2011). However, several authors have demonstrated a prolonged QTc interval duration in athletes as an uncommon observation, and QTc prolongation was considered to be unrelated to sports activities (Basavarajaiah et al., 2007). These data correspond to our findings, since native QT values were significantly longer in the running rabbits, although the difference disappeared when QT intervals were corrected to heart rate (QTc).

The T_peak_-T_end_ could forecast the development of TdP in LV wedge preparations (Fish et al., 2004) and in patients with LQT syndrome (Yamaguchi et al., 2003). Our experiments showed that long-term exercise significantly increased the duration of the T_peak_-T_end_ interval *in vivo*, which may imply an increased proarrhythmic sensitivity of the exercised animals. Interestingly, the T_peak_-T_end_ interval did not differ significantly between the “Ex” and “Sed” groups in Langendorff hearts, which indicates that the exercise-induced prolongation of the T_peak_-T_end_ interval seen *in vivo* was caused by either bradycardia or activity of the autonomic nervous system.

Results of human investigations and *in vivo* and *in vitro* animal experiments (Hinterseer et al., 2010) pointed out that repolarization BVI parameters increase during electrical remodeling. Lengyel et al. (2011) found increased STV QT values in competitive soccer players raising the possibility of an increased propensity for ventricular arrhythmias in that population. In our study, BVI parameters of QT intervals tended to be increased in the exercised group *in vivo*. Moreover, BVI of T_peak_-T_end_ intervals was significantly increased in the running rabbits *in vivo*, indicating altered repolarization due to long-term exercise.

Higher instability (ECG BVI parameter) and increased triangulation increase the proarrhythmic activity of drugs in isolated rabbit hearts even in the presence of lengthened or shortened ADP (Hondeghem et al., 2001). Thus, these parameters are regarded as proarrhythmia biomarkers. APD and triangulation were significantly prolonged in low K^+^ conditions in exercised hearts in our study. Exercise-induced hypokalemia was observed after strenuous exercise (Lindinger, 1995; Atanasovska et al., 2018). Hypokalemia is generally thought to be due to the reuptake of potassium into the muscle after exercise perhaps as the result of the continuation of catecholamine stimulation of the sarcolemmal sodium/potassium ATPase without anaerobic metabolism or muscle ischemia. Hypokalemia can be more serious if the electrolyte supplementation was not adequate or use of diuretics (e.g., for hypertension treatment). Hypokalemia, particularly when acute, contributes to the direct suppression of cardiac K^+^ channels and to the activation of Na^+^ and Ca^2+^ channels that contribute to impaired repolarization (Podrid, 1990; Weiss et al., 2017). Our results show that APD can lengthen, and triangulation may develop under hypokalemia in the myocardium after long-term exercise, which may have a role in arrhythmia development as a substrate.

Higher arrhythmic beat activity in the presence of I_Kr_ inhibition showed higher repolarization sensitivity in our model. The higher arrhythmic beat activity might provide triggers for re-entrant tachyarrhythmia, e.g., polymorphic tachycardia, under various conditions (Farkas and Nattel, 2010).

### Limitation of Our Study

It has to be mentioned that our study has some limitations. First, rabbits do not tolerate well heavy exercise, and accordingly the exercise-induced changes in our study were rather modest. In addition, QTc measurements in rabbit are rather uncertain since rabbit has distinctly different frequency-dependent repolarization relations than those of human (Arpadffy-Lovas et al., 2020), which may cause problems to accurately detect small alterations. As rabbit myocardium differs from the human in terms of ionic channel profile (e.g., lower expression of I_Ks_ in rabbits) (Lu et al., 2002; Dumaine and Cordeiro, 2007), electrophysiological remodeling might have different effects on repolarization in humans. However, it should be kept in mind that rigorous endurance training sometimes fails to produce pronounced electrophysiological effects in humans and the enhanced proarrhythmic risk is only evident if other predisposing factors, such as HCM, hypokalemia, doping, or QT-prolonging drugs are present. The Langendorff protocol may influence the dry weight of the heart and ventricles due to edema. Male gender dominance in the experimental groups might influence the severity of the structural, functional, and electrophysiological remodeling. As the mRNA level does not always reflect the protein level, different protein levels as compared with the mRNA levels cannot be excluded.

## Conclusion

In the present study, we extended our preliminarily published novel rabbit exercise-induced athlete’s heart model (Polyak et al., 2018). The detected echocardiography changes and signs of increased parasympathetic activity resemble those of the human athlete’s heart. The detected structural and functional changes in exercised rabbit hearts, e.g., increased fibrotic gene expression and alterations in repolarization, may directly contribute to the higher risk of the development of life-threatening arrhythmias in athletes, and the question that how enhanced vagal tone can contribute to the repolarization changes and possible enhanced proarrhythmic risk after heavy endurance training still needs further investigations.

## Data Availability Statement

The original contributions presented in the study are included in the article/Supplementary Material, further inquiries can be directed to the corresponding author/s.

## Ethics Statement

All procedures using animals were approved by the local ethics committee (including the Ethical Committee for the Protection of Animals in Research at the University of Szeged, Hungary) and conformed to the rules and principles of the 86/609/EEC Directive.

## Author Contributions

PK and HT performed the treadmill experiments and data collection. AP and BÖ performed the mRNA experiments. NN performed the APD experiments. LT managed the histology. IL, ArF, JP, and NJ managed the project and prepared the manuscript. AV, IB, and ASF performed the data collection, project management, and manuscript preparation. All authors contributed to the article and approved the submitted version.

## Conflict of Interest

The authors declare that the research was conducted in the absence of any commercial or financial relationships that could be construed as a potential conflict of interest.

## Publisher’s Note

All claims expressed in this article are solely those of the authors and do not necessarily represent those of their affiliated organizations, or those of the publisher, the editors and the reviewers. Any product that may be evaluated in this article, or claim that may be made by its manufacturer, is not guaranteed or endorsed by the publisher.
