# Supplementary material for: Long-Term Endurance Exercise Training Alters Repolarization in a New Rabbit Athlete’s Heart Model
Source: Front Physiol. 2022 Feb 14;12:741317. doi: 10.3389/fphys.2021.741317 (PMC8882986; doi:10.3389/fphys.2021.741317)
Supplement: Supplementary file 1 [file Table_1.DOCX]

| **Table 1.** | **Echocardiography parameters** | | | | | |
| --- | --- | --- | --- | --- | --- | --- |
|  | **Before training protocol** | | | **After training protocol** | | |
|  | ‘Sedentary’  group | ‘Exercised’  group | p value | ‘Sedentary’  group | ‘Exercised’  group | p value |
| **IVSd, mm** | 3.28±0.16 | 3.18±0.15 | 0.646 | 3.24±0.21 | 3.03±0.16 | 0.437 |
| **IVSs, mm** | 4.69±0.15 | 4.79±0.05 | 0.525 | 4.75±0.23 | 4.20±0.24 | 0.129 |
| **LVIDd, mm** | 14.70±0.54 | 15.80±0.32 | 0.104 | 14.44±0.62 | 17.25±0.31 | **0.002*** |
| **LVIDs, mm** | 10.58±0.36 | 10.84±0.21 | 0.537 | 10.57±0.58 | 11.81±0.29 | 0.078 |
| **LVPWd, mm** | 3.13±0.13 | 3.07±0.13 | 0.141 | 3.13±0.20 | 2.94±0.13 | 0.447 |
| **LVPWs, mm** | 4.74±0.24 | 4.95±0.27 | 0.577 | 4.59±0.22 | 4.96±0.42 | 0.447 |
| **Ao, mm** | 8.64±0.29 | 9.02±0.28 | 0.374 | 7.98±0.16 | 9.09±0.41 | **0.027*** |
| **EF, %** | 64.29±3.09 | 61.57±1.85 | 0.466 | 57.43±3.17 | 64.29±2.47 | 0.113 |
| **FS, %** | 32.43±2.42 | 30.43±1.25 | 0.477 | 27,86±2.04 | 32.71±1.7 | 0.092 |

**Table 1**-The effect of exercise training on echocardiographic cardiac dimensions and performance Values were measured before (at 0_th_ week, control measurements) and after (at 12^th^ week) the training protocol. IVSs and IVSd, Interventricular septum in systole and diastole; LVIDs and LVIDd, Left ventricular internal diameter in systole and diastole; LVPWs and LVPWd, Left ventricular posterior wall in systole and diastole; Ao, Aortic root diameter; EF, Ejection fraction; FS, Fractional shortening. All values are means±SEM. *P<0.05 vs. ’Sedentary’.
