# Supplementary material for: Long-Term Endurance Exercise Training Alters Repolarization in a New Rabbit Athlete’s Heart Model
Source: Front Physiol. 2022 Feb 14;12:741317. doi: 10.3389/fphys.2021.741317 (PMC8882986; doi:10.3389/fphys.2021.741317)
Supplement: Supplementary file 2 [file Table_2.DOCX]

| **Table 2.** | **Heart rate variability parameters** | | | | | |
| --- | --- | --- | --- | --- | --- | --- |
|  | ***In vivo*** | | | ***In vitro*** | | |
|  | ‘Sedentary’  group | ‘Exercised’  group | p value | ‘Sedentary’  group | ‘Exercised’  group | p value |
| **Mean_RR_, ms** | 265.5±5.7 | 324.5±10.5 | **0.001*** | 354.2±17.9 | 352.5±8.3 | 0.930 |
| **SD_RR_, ms** | 1.5±0.2 | 4.4±0.8 | **0.026*** | 1.6±0.7 | 3.4±1.7 | 0.360 |
| **RMS_RR_, ms** | 265.5±5.7 | 324.5±10.5 | **0.001*** | 354.2±17.9 | 352.5±8.3 | 0.931 |
| **rmsSD_RR_, ms** | 1.4±0.2 | 5.2±1.4 | **0.035*** | 2.2±1.2 | 5.0±2.8 | 0.401 |
| **sdSD_RR_, ms** | 1.5±0.2 | 5.2±1.4 | **0.047*** | 2.2±1.2 | 5.1±2.8 | 0.401 |
| **STV_RR_, ms** | 0.7±0.1 | 2.2±0.5 | **0.016*** | 0.7±0.2 | 2.0±0.9 | 0.199 |
| **LTV_RR_, ms** | 1.4±0.2 | 3.4±0.8 | **0.049*** | 1.0±0.3 | 2.0±0.8 | 0.317 |
| **TI_RR_, ms** | 1.8±0.2 | 4.1±1.0 | **0.047*** | 1.0±0.2 | 2.3±0.5 | **0.032*** |

**Table 2.** Heart rate variability parameters *in vivo* and *in vitro*. RMS, Root mean square; rmsSD, Root mean square of successive differences; sdSD, standard deviation of successive differences; STV, Short-term variability; LTV, Long-term variability; TI, Total instability; All values are means±SEM. *P<0.05 vs. ’Sedentary’.
