# Supplementary material for: Long-Term Endurance Exercise Training Alters Repolarization in a New Rabbit Athlete’s Heart Model
Source: Front Physiol. 2022 Feb 14;12:741317. doi: 10.3389/fphys.2021.741317 (PMC8882986; doi:10.3389/fphys.2021.741317)
Supplement: Supplementary file 3 [file Data_Sheet_1.DOCX]

**Supplement**

**Calculation methods and formula for the applied beat-to-beat variability of ECG intervals:**

RMS: the ‘root mean square’ (RMS) was calculated according to the following definition: $\text{RMS }=\text{ }\sqrt{\frac{1}{N}\sum_{i=0}^{N-1} d_{i}^{2}},$ where $d_{i}$ represents the sequence of RR or QT interval durations and $N$ is the total number of intervals.

RMSSD: Another approach for characterizing the beat-to-beat variability of ECG intervals is to take their successive differences ($\Delta d_{j}=d_{j+1}-d_{j}; 0\leq j\leq N-2,$ where $d_{j}$ represents the ECG interval durations and $N$is the total number of intervals) and calculate the root mean square of these differences: $\text{RMSSD}=\sqrt{E([\Delta d]^{2})},$ where $E$ denotes the mean value.

SDSD: the standard deviation of successive differences of the beat-to-beat variability of ECG intervals (Brennan et al., 2001).

### Instability

According to (Hondeghem et al., 2001), the instability of the RR and QT intervals was calculated as the difference between the upper quartile (the upper boundary of the lowest 75% of the interval values) and the lower quartile (the upper boundary of the lowest 25% of the interval values).

### Short-term variability (STV)

In terms of a Poincaré plot, which is a plot of RR or QT intervals (di+1) against the preceding RR or QT values (di), the short-term variability, introduced by (Thomsen et al., 2004), can be visualized as the mean perpendicular distance between the points of the plot and the di+1= di line.

### Long-term variability (LTV)

In the framework outlined above, long-term variability is the mean distance (measured parallel to the line di+1= di in the Poincaré plot) between the individual RR or QT interval durations (di) and their mean value (E(d)) (Thomsen et al., 2004).

### Echocardiography analysis:

Two-dimensional, M-mode and Doppler echocardiographic examinations were performed in accordance with the criteria of the American Society of Echocardiography. The mean values of three measurements were calculated and used for statistical evaluation. Systolic and diastolic wall thickness parameters were obtained from parasternal short-axis view at the level of the papillary muscles and long-axis view at the level of the mitral valve. The left ventricle diameters were measured by means of M-mode echocardiography from long-axis and short-axis views between the endocardial borders. Echocardiography parameters were analysed by an investigator in a randomised and blinded manner. The investigator does not know if the animal participated in the training or not. Moreover, the investigations were randomized to prevent bias and potential harmful effects influencing the measurements. The applied ECHO device using a built-in software package calculates the fractional shortening and ejection fraction with the following formulas:

### Fractional shortening = [(LVIDd-LVIDs)/LVIDd] × 100.

### Teicholz method was apllied for the ejection fraction:

### Vd = [7/(2.4+LVIDd)] x LVIDd^3^

### Vs = [7/(2.4+LVIDs)] x LVIDs^3^

### Left ventricular ejection fraction = (Vd-Vs)/Vd

Vd: End-diastolic volume, Vs: End-systolic volume; LVIDd: left ventricular internal diameter in diastole; LVIDs: left ventricular internal diameter in systole

**Langendorff-perfusion protocol**

The Langendorff-perfusion protocol applied at the end of 12-week long training protocol. *In vivo* echocardiography and ECG measurements preceded the isolated heart experiments. The weight of heart and chambers were measured after the completion of Langendorff perfusion experiments.

**References:**

Brennan, M., Palaniswami, M., and Kamen, P. (2001). Do existing measures of Poincare plot geometry reflect nonlinear features of heart rate variability? *IEEE Trans Biomed Eng* 48(11)**,** 1342-1347. doi: 10.1109/10.959330.

Hondeghem, L.M., Carlsson, L., and Duker, G. (2001). Instability and triangulation of the action potential predict serious proarrhythmia, but action potential duration prolongation is antiarrhythmic. *Circulation* 103(15)**,** 2004-2013.

Thomsen, M.B., Verduyn, S.C., Stengl, M., Beekman, J.D., de Pater, G., van Opstal, J., et al. (2004). Increased short-term variability of repolarization predicts d-sotalol-induced torsades de pointes in dogs. *Circulation* 110(16)**,** 2453-2459. doi: 10.1161/01.CIR.0000145162.64183.C8.
